# The Challenge of Modeling the Acquisition of Mathematical Concepts

**DOI:** 10.3389/fnhum.2020.00100

**Published:** 2020-03-20

**Authors:** Alberto Testolin

**Affiliations:** ^1^Department of General Psychology, University of Padova, Padova, Italy; ^2^Department of Information Engineering, University of Padova, Padova, Italy

**Keywords:** computational modeling, artificial neural networks, deep learning, number sense, symbol grounding, mathematical learning, embodied cognition, material culture

## Abstract

As a full-blown research topic, numerical cognition is investigated by a variety of disciplines including cognitive science, developmental and educational psychology, linguistics, anthropology and, more recently, biology and neuroscience. However, despite the great progress achieved by such a broad and diversified scientific inquiry, we are still lacking a comprehensive theory that could explain how numerical concepts are learned by the human brain. In this perspective, I argue that computer simulation should have a primary role in filling this gap because it allows identifying the finer-grained computational mechanisms underlying complex behavior and cognition. Modeling efforts will be most effective if carried out at cross-disciplinary intersections, as attested by the recent success in simulating human cognition using techniques developed in the fields of artificial intelligence and machine learning. In this respect, deep learning models have provided valuable insights into our most basic quantification abilities, showing how numerosity perception could emerge in multi-layered neural networks that learn the statistical structure of their visual environment. Nevertheless, this modeling approach has not yet scaled to more sophisticated cognitive skills that are foundational to higher-level mathematical thinking, such as those involving the use of symbolic numbers and arithmetic principles. I will discuss promising directions to push deep learning into this uncharted territory. If successful, such endeavor would allow simulating the acquisition of numerical concepts in its full complexity, guiding empirical investigation on the richest soil and possibly offering far-reaching implications for educational practice.

## Introduction

Despite the importance of mathematics in modern societies, the cognitive foundations of mathematical learning are still mysterious and hotly debated. At the one end of the bridge, the idealistic view conceives mathematical concepts as purely abstract entities that humans discover using logical reasoning; at the other end, empiricists argue that mathematics is the product of our sensory experiences, and therefore it is essentially an activity of construction (Brown, 2012). A somehow intermediate position is taken by modern neurocognitive theories, which identify a set of “core” brain systems specifically evolved to support basic intuitions about quantity (Butterworth, 1999; Feigenson et al., 2004; Piazza, 2010; Dehaene, 2011) but also acknowledge that higher-level numerical knowledge has materialized only recently, via cultural practices supported by language and symbolic reference (Núñez, 2017).

In recent years, the finding that measures of basic quantification skills correlate to later mathematical achievement (e.g., Halberda et al., 2008; Libertus et al., 2011; Starr et al., 2013) has led to the hypothesis that our “number sense” might indeed constitute the starting point to learn more complex mathematical concepts. However, the relationship between numerosity perception and symbolic math remains controversial (Negen and Sarnecka, 2015; Schneider et al., 2017; Wilkey and Ansari, 2019), calling for a deeper theoretical investigation that should be carried out with the support of formal models.

Here I will argue that the quest for artificial intelligence provides an extremely rich soil for the development of a computational theory of mathematical learning. Indeed, although computers largely outperform humans on numerical tasks requiring the mere application of syntactic manipulations (e.g., performing algebraic operations on large numbers, or iteratively computing the value of a function), they are completely blind about the *meaning* of such operations because they lack a conceptual semantics of number. Grounding abstract symbols into some form of intrinsic meaning is a longstanding issue in artificial intelligence (Searle, 1980; Harnad, 1990), and mathematics likely constitutes the most challenging domain for investigating how high-level knowledge could be linked to bottom-up, sensorimotor primitives (Leibovich and Ansari, 2016).

By framing a theory in computational terms, scientists are forced to adopt a precise, formal language, because all the details of the theory should be explicitly stated to simulate it on a computer. Modeling also requires to carefully think about the tasks that are being simulated and the possible ways in which a computational device can (or cannot) solve them. In this perspective article, I will focus in particular on *connectionist* models, where cognition is conceived as an emergent property of networks of units that self-organize according to physical principles (Rumelhart and McClelland, 1986; Elman et al., 1996; McClelland et al., 2010). According to this view, knowledge is implicitly stored in the connections among neurons, and learning processes adaptively change the strength of these connections according to experience. Notably, the recent breakthroughs in *deep learning* (LeCun et al., 2015) have revealed the true potential of this approach, by showing how machines endowed with domain-general learning mechanisms can simulate a variety of high-level cognitive skills, ranging from visual object recognition (He et al., 2016) to natural language understanding (Devlin et al., 2018) and strategic planning (Silver et al., 2017).

### Computational Models of Basic Quantification Skills

**Figure 1:**
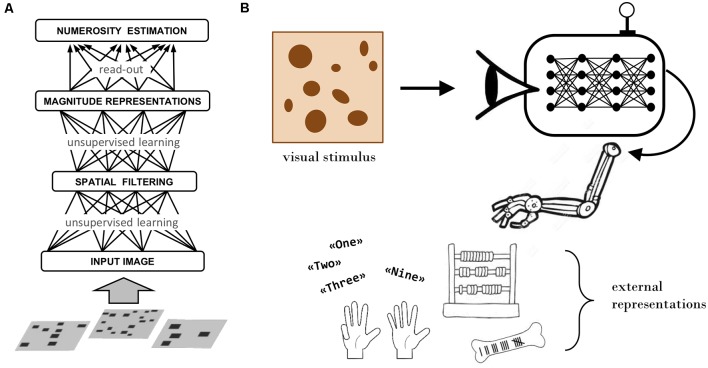
Deep learning models. **(A)** Schematic representation of an unsupervised deep learning model that simulates human numerosity perception. Adapted from Zorzi and Testolin (2018). **(B)** Sketch of the proposed modeling framework, which extends the basic numerosity perception model (entirely confined within the agent’s brain) by introducing the ability to interact with the external environment to create and manipulate material representations.

According to the “number sense” view, numerical cognition is grounded in basic quantification skills, such as the ability to rapidly estimate the number of items in a visual display (Dehaene, 2011). Numerosity is thus conceived as a primary perceptual attribute (Anobile et al., 2016) processed by a specialized (and possibly innate) system yielding an approximate representation of numerical quantity (Feigenson et al., 2004). The seminal neural network model by Dehaene and Changeux (1993) incorporated these principles: numerosity perception was hardwired in the model, reflecting the assumption that this ability is present at birth. Successive models revisited this nativist stance, by showing that numerosity representations can emerge as a result of learning and sensory experience (Verguts and Fias, 2004). In particular, recent work based on unsupervised deep learning has demonstrated that human-like numerosity perception can emerge in multi-layer neural networks that learn a hierarchical generative model of the sensory data (Stoianov and Zorzi, 2012; Zorzi and Testolin, 2018; see Figure 1A).

Deep learning models account for a wide range of empirical phenomena in the number sense literature. They can accurately simulate Weber-like responses in numerosity comparison tasks (Stoianov and Zorzi, 2012), also accounting for congruency effects (Zorzi and Testolin, 2018) and for the fine-grained contribution of non-numerical magnitudes in biasing behavioral responses (Testolin et al., 2019). Notably, the number acuity of randomly initialized deep networks rivals that of newborns, and its gradual development follows trajectories similar to those observed in human longitudinal studies (Testolin et al., 2020). Deep networks have also been successfully tested in subitizing (Wever and Runia, 2019) and numerosity estimation tasks (Chen et al., 2018). Last, but not least, artificial neurons often reproduce neurophysiological properties observed in single-cell recording studies, for example by exhibiting number-sensitive tuning functions (Zorzi and Testolin, 2018; Nasr et al., 2019).

Several questions remain under investigation: Is it possible to fully disentangle numerosity from continuous magnitudes by only relying on unsupervised learning (Zanetti et al., 2019)? Can generative models generalize to unseen numerosities (Zhao et al., 2018)? Are there computational limitations in tracking multiple objects in dynamic scenes (Cenzato et al., 2019)? What is the contribution of explicit feedback and multi-sensory integration in shaping numerosity representations? How do deep learning models map into the cortical processing hierarchy? Nevertheless, despite these open questions, we can safely argue that deep learning has paved the way toward a computational theory about the origin of our number sense, confirming the appeal of deep networks as models of human sensory processing (Testolin and Zorzi, 2016; Yamins and DiCarlo, 2016; Testolin et al., 2017). Unfortunately, simulating the transition from approximate to symbolic numbers turns out to be much more challenging, as we discuss in the next section.

### Modeling the Acquisition of Higher-Level Mathematical Concepts

One of the most ambitious questions to be addressed is whether deep learning models could develop even more sophisticated numerical abilities, such as those involving arithmetic and symbolic math. Symbolic reasoning is notoriously difficult for connectionist models (Marcus, 2003), and despite recent progress, deep neural networks still struggle with tasks requiring procedural and compositional knowledge (Garnelo and Shanahan, 2019).

Only a few modeling studies have investigated how arithmetic could be learned by artificial neural networks. Since early attempts, associative memories have been used to simulate mental calculation as a process of storage and retrieval of arithmetic facts (McCloskey and Lindemann, 1992): during the learning phase, the two arguments and the result of a simple operation (e.g., single-digit multiplication) are given as input to an associative memory, whose learning goal is to accurately store them as a global, stable state. During the testing phase, only the operands are given, and the network must recover the missing information (i.e., the result) by gradually settling into the correct configuration. Building on this approach, successive simulations have shown that numerosity-based (“semantic”) representations can facilitate the learning of arithmetic facts (Zorzi et al., 2005) and equivalence problems (Mickey and McClelland, 2014). Others have shown that multi-digit addition and subtraction (but not multiplication) can be acquired through end-to-end supervised learning from pixel-level images (Hoshen and Peleg, 2015). One critical limitation of these approaches, however, is that they conceive arithmetic learning as a mere process of storing and recall, which gradually develops through the massive reiteration of all possible arithmetic facts that need to be learned. Besides being psychologically implausible and computationally unfeasible, this approach does not guarantee that the system will be able to generalize the acquired knowledge to unseen numbers and, even less, to exploit the acquired knowledge to more effectively learn new mathematical concepts.

The challenge of developing learning models that can exhibit algebraic generalization with the robustness and flexibility exhibited by humans is so fundamental that major players in deep learning research are intensively investigating these issues. For example, Google’s DeepMind company has recently evaluated several deep learning models on a set of benchmark problems taken from UK national school mathematics curriculums, covering arithmetic, algebra, elementary calculus, et cetera (Saxton et al., 2019). DeepMind’s best model correctly solved only 14 out of 40 problems, which would be equivalent to an “E” grade. Although such difficulties have led some researchers to argue that neural networks are incapable of exhibiting compositional abilities (Marcus, 2018), others argue for the opposite (Baroni, 2020; Martin and Baggio, 2020).

Even the acquisition of the concept of *exact number* is still out of reach for deep networks, which often cannot generalize outside of the range of numerical values encountered during training (Trask et al., 2018). Integer numbers are one of the pillars of arithmetic, so they constitute the perfect testbed for developing and testing computational models of mathematical learning. Developmental studies show that integers are gradually acquired by children during formal education through the acquisition of number words and counting skills: Indeed, although sequential (item-by-item) enumeration skills are present in animal species (Platt and Johnson, 1971; Beran and Beran, 2004; Dacke and Srinivasan, 2008), even in humans counting is not culturally universal (Gordon, 2004) and there is evidence that young children and people from cultures lacking number words have an incomplete understanding of what it means for two sets of items to have exactly the same number of items (Izard et al., 2008, 2014).

Some authors have sought to characterize the acquisition of exact numbers as the semantic induction of a “cardinality principle” (Sarnecka and Carey, 2008). This hypothesis has been exemplified in a computational model based on Bayesian inference, which simulated the stage-like development of counting abilities by relying on a pre-determined set of “core” cognitive operations (Piantadosi et al., 2012). The repertoire of innate abilities included the capacity to exactly identify cardinalities up to 3, perform basic operations on sets (e.g., difference, union, intersection), retrieve the next or previous word from an ordered counting list, and to operate these functions recursively. Although such modeling approach offers a rational interpretation of the process that might underly the acquisition of an abstract cardinality principle, it assumes a certain amount of *a priori* symbolic knowledge and procedural skills, which is in contrast to empirical data suggesting, for example, that a complete understanding of the successor principle arises only after considerable interaction with the teaching environment (Davidson et al., 2012).

## Toward a Comprehensive Neurocomputational Framework

### The Downplayed Role of External Representations

A central tenet of connectionist models is that semantics intrinsically emerges in a system interacting with its surrounding environment. However, this idea is usually superficially implemented in deep learning models, because the interaction is often limited to *passive* observation of statistical properties of the world (Zorzi et al., 2013). Taking inspiration from constructivist theories in developmental psychology, here I argue that a step forward will require to build computational models that learn by *actively* manipulating the environment, that is, by causally interacting with objects in their perceptual space. Crucially, the notion of “environment” should include embodiment (Lakoff and Núñez, 2000) and—most importantly—the social, cultural and educational environment (Vygotsky, 1980; Clark, 2011). Indeed, according to the Vygotskyan perspective, students actively construct abstract knowledge through interactions with teachers and peers, gradually moving their dependency on explicit forms of mediation to more implicit (internalized) forms (Walshaw, 2017).

The possibility to manipulate the environment greatly increases the complexity of the learning agent but also enables the functional use of external entities to create powerful representational systems, which can be manipulated in simple ways to get answers to difficult problems. The underlying assumption is that cultural evolution and history are foundational forces for the emergence of superior cognitive functions and that great intellectual achievements (such as the invention of mathematics) have been triggered by our ability to create artifacts serving as physical representations of abstract concepts. Some investigators have recently emphasized the role of material culture in numerical cognition (Menary, 2015; Overmann, 2016, 2018), for example by highlighting that our mental organization of numbers into an ordered “number line” might be related to the linearity of the material forms used to represent and manipulate them (Núñez, 2011). Primitive devices used for representing numbers date back to notched bones in the Paleolithic period (d’Errico et al., 2018) and clay tokens in the Neolithic period (Schmandt-Besserat, 1992), which predated the subsequent diffusion of abaci, positional systems and increasingly more sophisticated numerical notations (Menninger, 1992). However, despite the concept of external representations was foreseen in early connectionist theories^1^, it has been seldomly explored in practice.

### Learning to Create and Manipulate Symbolic Representations

We can now sketch a concrete proposal for building more realistic simulations of mathematical learning. The computational framework should incorporate the following key components, summarized in Figure 1B.

- **Perceptual system**. This is where computational modeling has been mostly focused (and successful) up to now (see Section “Computational Models of Basic Quantification Skills”). The challenge will be to scale-up the existing models to more realistic sensory input (e.g., naturalistic visual scenes) and to incorporate a larger repertoire of pattern recognition abilities, which should not only allow to approximately represent visual quantities but also to recognize structured configurations of object arrays (e.g., sequences of tally marks, geometric displacements of items, patterns encoded in an abacus, etc.) and symbolic notations (e.g., written digits and operands).
- **Embodiment**. Of particular interest to the development of exact numbers is finger counting (Butterworth, 1999; Andres et al., 2007; Domahs et al., 2012), which not only helps children to keep track and coordinate the production of number words (Alibali and DiRusso, 1999) but may also allow to organize numbers spatially (Fischer, 2008). Hand-based representations are ubiquitous across cultures (Bender and Beller, 2012) and play a key role in the subsequent acquisition of number words (Gunderson et al., 2015; Gibson et al., 2019), possibly influencing symbolic number processing even in adulthood (Domahs et al., 2010). It has been recently shown that neural networks can learn to count the number of items in visual displays and that the ability to sequentially point to individual objects helps in speeding up counting acquisition (Fang et al., 2018). A further step is taken by cognitive developmental robotics, which explores the instantiation of these principles in physically embodied agents (Di Nuovo and Jay, 2019). Interestingly, pointing gestures significantly improved counting accuracy in a humanoid robot, and learning was more effective when both fingers and words were provided as input (Rucinski et al., 2012; De La Cruz et al., 2014).
- **Material representations**. The ability to manipulate external objects might be the key missing piece for simulating the acquisition of exact numbers. Indeed, although hand gestures might serve as placeholders to learn more efficient arithmetic strategies (Siegler and Jenkins, 1989; for a computational account see Hansen et al., 2014), material representations allow for a much more precise encoding of numerical information. For example, the agent can learn to establish the cardinality of a set by organizing items in regular configurations that promote “groupitizing” (Starkey and McCandliss, 2014), or to exactly compare the cardinality of two sets by disposing of items in one-to-one correspondence. More sophisticated devices such as abaci and Cuisenaire rods further extend our ability to represent exact numbers, for example by exploiting inter-exponential relations to precisely (but compactly) encode large numbers, or to explicitly represent compositionality to promote generalization (Overmann, 2018).
- **Diversified learning signals**. In addition to unsupervised learning, the agent should exploit *reinforcement learning* (Sutton and Barto, 1998) to predict the outcome of its actions. This learning modality would also play a key role in simulating curiosity-driven behavior and active engagement with material representations. Notably, deep reinforcement learning has recently achieved impressive performance in difficult cognitive tasks, for example by discovering complex strategies in board games (Silver et al., 2017). However, learning through reinforcement can be challenging in the presence of very large action spaces (i.e., the correct action has to be chosen from a wide range of possible actions) and sparse rewards (i.e., feedback is given only once the whole task has been carried out). Taking inspiration from the notions of *transfer learning* and *curriculum learning* used in machine learning (Bengio et al., 2009) and from *shaping* procedures used in animal conditioning (Skinner, 1953), these issues can be mitigated by decomposing the task into simpler sub-tasks. For example, rather than rewarding only the trials where the agent has correctly counted all items in a display, rewards can be initially given every time the agent touches an object, to first promote the acquisition of sequential pointing skills. Similarly, the agent could first be rewarded simply for being able to accurately reproduce the abacus configuration corresponding to a specific number, rather than for being able to correctly manipulate the abacus to solve an addition problem. This idea of “gradually walking the agent through the word” also implies the exploitation of *supervised learning*, because explicit teaching signals must be used to stimulate learning by imitation and adult guidance.
- **Linguistic input**. Despite language might not be crucial for the acquisition of elementary numerical concepts (Gelman and Butterworth, 2005; Butterworth et al., 2008), it provides useful cues during the development of basic algebraic notions: for example, morphological cues allow single/plural distinction, number words can act as stable placeholders during counting acquisition, and learning natural language quantifiers seems a key step for mastering the ordering principle (Le Corre, 2014). A recent deep learning model has shown that learning quantifiers allows to more easily carry out approximate numerosity judgments (Pezzelle et al., 2018); however, the role of linguistic input for simulating the acquisition of exact numbers has yet to be explored. Furthermore, later in development language becomes the primary medium to acquire higher-level mathematical knowledge, hence it will need to be taken into account to design computational models approaching that level of complexity.

## Discussion

Symbolic numbers are a hallmark of human intelligence, but we are still lacking a comprehensive theory explaining how the brain learns to master them. Here I argued that computational modeling should have a primary role in this enterprise. Taking the acquisition of natural numbers as a case study, I emphasized the role of material representations in supporting the transition from approximate to symbolic numerical concepts. According to this view, exact numbers do not emerge from the mere association between number words and perceptual magnitudes: such mapping is strongly mediated by the acquisition of procedural skills (e.g., finger counting) and the ability to effectively manipulate representational devices (Leibovich and Ansari, 2016; Overmann, 2018; Carey and Barner, 2019).

**Figure 2:**
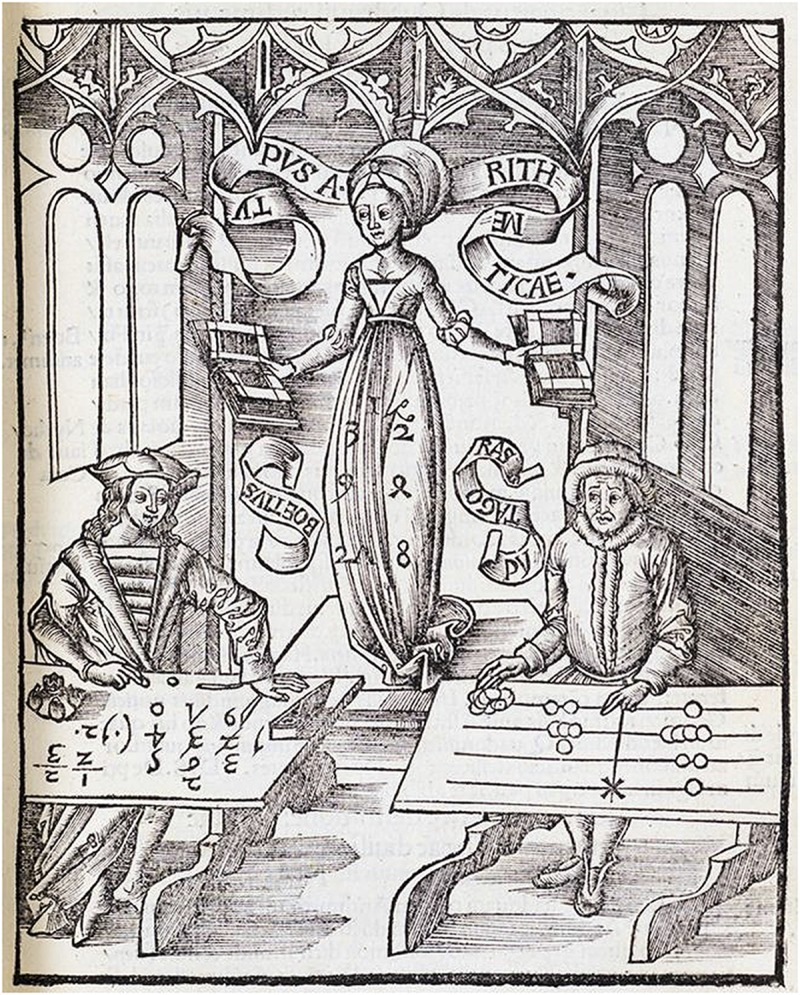
Allegory of Arithmetic. Engraving from the encyclopedic book *Margarita Philosophica* by Gregor Reisch (1503) depicting the “abacists vs. algorists” debate. Arithmetica (female figure) is supervising a calculation contest between Pythagoras (right), represented as using a counting board, and Boethius (left), who embraces algorithmic calculation with Arabic numbers. The struggle of Pythagoras suggests who is going to be the winner. Reproduced from Wikipedia.

In line with the idea that improved problem representation is a key mechanism for the joint development of conceptual and procedural knowledge (Rittle-Johnson et al., 2001), cognitive development in artificial agents must thus be supported by an adequate learning environment, which should provide feedback, teaching signals, and representational media commensurate with the current level of development. Notably, once a procedural skill has been mastered it might become internalized: the agent can simply “imagine” carrying out operations on the material device, without the need to physically operate over it. Some representations might thus serve just as intermediate steps for the acquisition of more abstract and efficient notations: as finger counting allows us to gradually grasp the meaning of number words, manipulating an abacus allows to ground numerical symbols into concrete visuospatial representations. A historical case that illustrates this perspective is the famous dispute between “abacists” and “algorists”, which was undoubtedly won by the latter, who demonstrated the superiority of symbolic notation for carrying out arithmetic operations (see Figure 2). However, one might wonder whether Boethius could have mastered arithmetic algorithms without first grounding his numerical concepts into a set of more concrete representations.

In addition to providing a useful framework to interpret empirical findings, the proposed approach can raise important questions that would stimulate further theoretical and experimental work. For example, a critical aspect of our school system is to teach how to effectively discover useful strategies and representational schemes for solving difficult problems. In computational simulations, the necessity for appropriate teacher guidance stems from the fact that it is very difficult to invent new representations for problems we might wish to solve: it may even be that the process of inventing such representations is one of our highest intellectual abilities (Rumelhart et al., 1986). Computational frameworks that allow simulating a more complex interaction between artificial agents and their learning environment might thus eventually provide insights also about the teaching practices that could be most effective to guide numerical development in our children.

## Author Contributions

AT is fully responsible for the content of this article.

## Conflict of Interest

The author declares that the research was conducted in the absence of any commercial or financial relationships that could be construed as a potential conflict of interest.

## Footnotes

^1^See for example, the section “External Representations and Formal Reasoning” in Rumelhart et al. (1986).
